# Identification, collection, and reporting of harms among non-industry-sponsored randomized clinical trials of pharmacologic interventions in the critically ill population: a systematic review

**DOI:** 10.1186/s13054-020-03113-z

**Published:** 2020-07-08

**Authors:** Ari Moskowitz, Lars W. Andersen, Mathias J. Holmberg, Anne V. Grossestreuer, Katherine M. Berg, Asger Granfeldt

**Affiliations:** 1 Division of Pulmonary, Critical Care, and Sleep Medicine, Beth Israel Deaconess Medical Center, One Deaconess Rd, W/CC 2, Boston, MA 02215 USA; 2 Research Center for Emergency Medicine, Aarhus University Hospital and Aarhus University, Aarhus, Denmark; 3 Prehospital Emergency Medical Services, Aarhus, Central Denmark Region Denmark; 4 Department of Emergency Medicine, Beth Israel Deaconess Medical Center, Boston, MA USA; 5 Department of Critical Care, Aarhus University Hospital, Aarhus, Denmark

**Keywords:** Clinical trial, Adverse event, Harm, CONSORT

## Abstract

**Background:**

Prescribing pharmacologic therapies for critically ill patients requires a careful balancing of risks and benefits. Defining, monitoring, and reporting harms that occur in clinical trials conducted in critically ill populations, however, is challenging given that the natural history of most critical illnesses includes progressive multiple organ failure and death. In this study, we assessed harms reporting in clinical trials performed in critically ill populations.

**Methods:**

Randomized, non-industry-sponsored, human clinical trials of pharmacologic interventions in adult critically ill populations published between 2015 and 2018 in high-impact journals were included in this systematic review. Harms data, adherence to Consolidated Standards of Reporting Trials (CONSORT) harms reporting guidelines, and restrictions on harms reporting were recorded.

**Results:**

A total of 707 abstracts were screened with 40 trials ultimately being included in the analysis. Included trials represent 28,636 randomized patients with a median of 292 (IQR 100–546) patients per trial. The most common disease states were general critical care (33%) and sepsis (28%). Of 18 included CONSORT items, the median number met was 12 (IQR 9, 14). The most commonly missed items were adverse event (AE) severity grading definitions and AE attribution (relationship of AE to study drug), which were only reported in 35 and 38% of manuscripts, respectively. Half of the manuscripts (48%) provided definitions for recorded AEs. There were 5 studies investigating the effects of corticosteroids in sepsis, with the number of AEs reported per analyzed patient ranging from 0.01 to 1.89. AE definitions in studies of similar/equivalent interventions often varied substantially. Study protocols were available for 30/40 (75%) of studies, with 13 (43%) of those not providing any guidance regarding AE attribution.

**Conclusions:**

Randomized trials of pharmacologic interventions conducted in critically ill populations and published in high impact journals often fail to adequately describe AE definitions, severity, attribution, and collection procedures. Among trials of similar interventions in comparable populations, variation in AE collection and reporting procedures is substantial. These factors may limit a clinician’s ability to accurately balance the potential benefits and harms of an intervention.

## Introduction

Prescribing pharmacologic therapies for critically ill patients requires a careful balancing of potential efficacy against potential harm. Randomized clinical trials (RCT) are a crucial research tool, which allow investigators and clinicians insight into the risk/benefit ratio of medical therapies and promote informed decision-making in the intensive care unit (ICU). The published results of clinical trials, however, frequently focus on the potential efficacy of the intervention with limited (if any) discussion of potential harms [1]. The reason for this is multifactorial and likely includes investigator excitement regarding the studied intervention and the difficulty in accurately identifying, collecting, and reporting adverse events (AEs; any adverse outcomes potentially related to the active intervention) [2]. The latter is particularly challenging in clinical trials conducted in critically ill populations where the distinction between a suspected adverse drug reaction (in which there is a reasonable possibility that the AE was related to the study drug) and the natural history of the critical illness is often difficult to determine [3]. To date, there have been no studies assessing the identification, collection, and reporting of harms in contemporary trials of critical care pharmacologic interventions.

In the present study, we assessed harms reporting in contemporary trials of pharmacologic critical care interventions. We measured adherence to CONSORT recommendations in the published manuscripts and reviewed the published protocols to allow a more detailed description of harms collection and assessment. Further, we assessed variability in harms collection and reporting among trials studying similar interventions.

## Methods

### Study design

The present study is a systematic review of harms reporting in critical care trials of pharmacologic interventions. The manuscript adheres to standard systematic review conventions; however, the Preferred Reporting Items for Systematic Reviews and Meta-Analyses (PRISMA) guidance does not directly apply given that the present study does not focus on specific interventions, but rather on harms reporting [4].

### Study eligibility

Randomized, non-industry-sponsored human clinical trials of pharmacologic interventions in adult critically ill populations published between January 1, 2015, and December 31, 2018, in high-impact medical journals were included. Only high-impact journals were selected to allow an efficient assessment of those trials perhaps more rigorously vetted by peers, and those with the greatest reach into clinical practice. Industry studies were excluded as they are more commonly focused on obtaining regulatory approval, often requiring extensive safety monitoring. Industry studies may also have more resources for safety monitoring, which is not representative of what is feasible for most academic investigators. Studies were included if they compared one specific pharmacologic therapy (either type, dosage, frequency, or duration) against another pharmacologic therapy or placebo. Only studies in which at least 25 patients per study arm were enrolled were considered. Critically ill patients were defined as patients evaluated in the emergency department or ICU setting who carried a high probability of imminent or life-threatening clinical deterioration as determined by the physician reviewers. High-impact medical journals included were (1) *New England Journal of Medicine* [NEJM], (2) *Lancet*, (3) *Journal of the American Medical Association* [JAMA], (4) *British Medical Journal* [BMJ], (5) *The American Journal of Respiratory and Critical Care Medicine* [AJRCCM], (6) *Critical Care Medicine* [CCM], (7) *Intensive Care Medicine* [ICM], and (8) *Chest*. These journals were selected by a consensus of the authors who are all critical care physicians and/or researchers.

### Information sources

The MEDLINE electronic bibliographic database was used to identify studies potentially meeting inclusion criteria. The search term can be found in the Supplementary Materials and focuses on critical care RCTs published in the above journals during the target time frame. In addition, each journal’s archives from the included years were manually reviewed to identify any RCTs not captured by the search strategy.

### Selection process

Two reviewers independently screened all titles and abstracts in duplicate. The reviewers were blinded to authors during this screening stage. Any disagreements regarding inclusion or exclusion were resolved via discussion between the reviewers. The level of agreement was measured via Cohen’s kappa and a *ƙ* < 0.60 was set as a limit at which point a 3rd reviewer would review all abstracts. After identifying those titles/abstracts for manuscripts potentially meeting all inclusion and no exclusion criteria, the two reviewers then independently reviewed all full-length manuscripts to identify a final list of studies for data abstraction.

### Clinical trial protocols

For each included trial, we attempted to obtain the clinical trial protocol. To this end, we searched clinical trial registries (e.g., ClinicalTrials.gov) and published protocols in peer-reviewed journals. If the protocol could not be obtained through these methods, the author/study group was contacted for additional information. If no protocol could be obtained, this is noted in the “Results” section.

### Data abstraction

A predefined standardized data abstraction form was used to extract data as pertinent to the research question. AEs were defined as any adverse outcomes potentially related to the active intervention reported, including any safety outcomes described by the investigators. Included studies were reviewed independently by two reviewers and disagreements with respect to data abstraction or harms reporting resolved via discussion between those reviewers. A third reviewer was available to adjudicate any substantial disagreement.

As has been done in prior studies when assessing adherence to CONSORT harms recommendations, a set of items derived from the 10 items in the 2004 CONSORT harms checklist was used. This derived set of items was based on previously used checklist criteria and modified by the authors to better reflect trials in the critical care setting [5, 6]. This step was taken to better operationalize the checklist and ensure homogeneity in recording, given the open-ended nature of the original checklist items. Item 9 on the CONSORT checklist was excluded as subgroup analyses for AEs are rare. A liberal approach was taken to the assessment of each criterion, and partial completion was generally considered satisfactory. The published manuscript, including any supplemental material, was reviewed. Information present in data supplements was considered equal to that in the manuscript. The operational checklist used for this study can be found in Table 1 and ultimately included 18 items [5–7].

**Table 1:** CONSORT checklist adherence

CONSORT harm checklist number	CONSORT item definitions	Operational items	Adherence rate, ***n*** (%)
1	If the study collected data on harms and benefits, the title or abstract should so state.	Adverse events (AEs) mentioned in the manuscript title or abstract	29 (73)
2	If the trial addresses both harms and benefits, the introduction should so state.	Information on AEs mentioned in the manuscript introduction	20 (50)
3	List addressed adverse events with definitions for each (with attention, when relevant, to grading, expected vs. unexpected events, reference to standardized and validated definitions, and description of new definitions).	The manuscript lists addressed AEs and provides definitions for each	19 (48)
		Manuscript mentions how AE severity grading was performed (e.g. any use of a validated scale)	14 (35)
4	Clarify how harms-related information was collected (mode of data collection, timing, attribution methods, intensity of ascertainment, and harms-related monitoring and stopping rules, if pertinent).	Manuscript describes how AE data were collected (e.g. real-time case review, post hoc review)	21 (53)
		Manuscript describes the time period over which AE surveillance occurred	22 (55)
		Manuscript reports whether AEs were related to study drug or not	15 (38)
		Manuscript describes how AE monitoring was performed (e.g., DSMB, trial monitor)	27 (68)
5	Describe plans for presenting and analyzing information on harms (including coding, handling of recurrent events, specification of timing issues, handling of continuous measures, and any statistical analyses).	Manuscript describes methods for presenting and/or analyzing AEs	26 (65)
6	Describe for each arm the participant withdrawals that are due to harms and their experiences with the allocated treatment.	Manuscript reports the number of withdrawals due to AEs in each arm. If withdrawals due to an AE occurred, a description of the AE is provided	23 (58)
		Manuscript reports the number of deaths due to AEs in each arm. If death due to an AE occurred, a description of the AE is provided	18 (45)
7	Provide the denominators for analyses on harms.	Manuscript reports the denominator used for AE analysis (i.e., total number of patients analyzed)	39 (99)
		The efficacy and safety analyses are performed using the same populations	36 (90)
8	Present the absolute risk per arm and per adverse event type, grade, and seriousness, and present appropriate metrics for recurrent events, continuous variables, and scale variables, whenever pertinent.	Safety results presented separately for each treatment arm	39 (98)
		Manuscript provides both the number of AEs and the number of patients with AEs	37 (93)
		Manuscript presents severity/grading of AEs	17 (43)
9	Describe any subgroup analyses and exploratory analyses for harms.	Not Included	N/A
10	Provide a balanced discussion of benefits and harms with emphasis on study limitations, generalizability, and other sources of information on harms.	Manuscript presents prior literature in the discussion in relation to AEs	22 (55)
		Discussion section presents a balanced view of risks and benefits	21 (53)

In cases where at least 2 RCTs examined the same or very similar intervention in a critically ill population, expected AE definitions and frequencies were directly compared.

### Statistical analysis

Adherence to CONSORT recommendations was assessed by determining the total number of criteria met in each trial. Two-way comparisons were made using Wilcoxon rank-sum tests or Fisher’s exact tests as appropriate. All statistical tests were two-sided and an alpha < 0.05 considered statistically significant.

StataCorp. 2019. *Stata Statistical Software: Release 16*. College Station, TX: StataCorp LLC was used for all analyses.

## Results

### Study selection

**Fig. 1:**
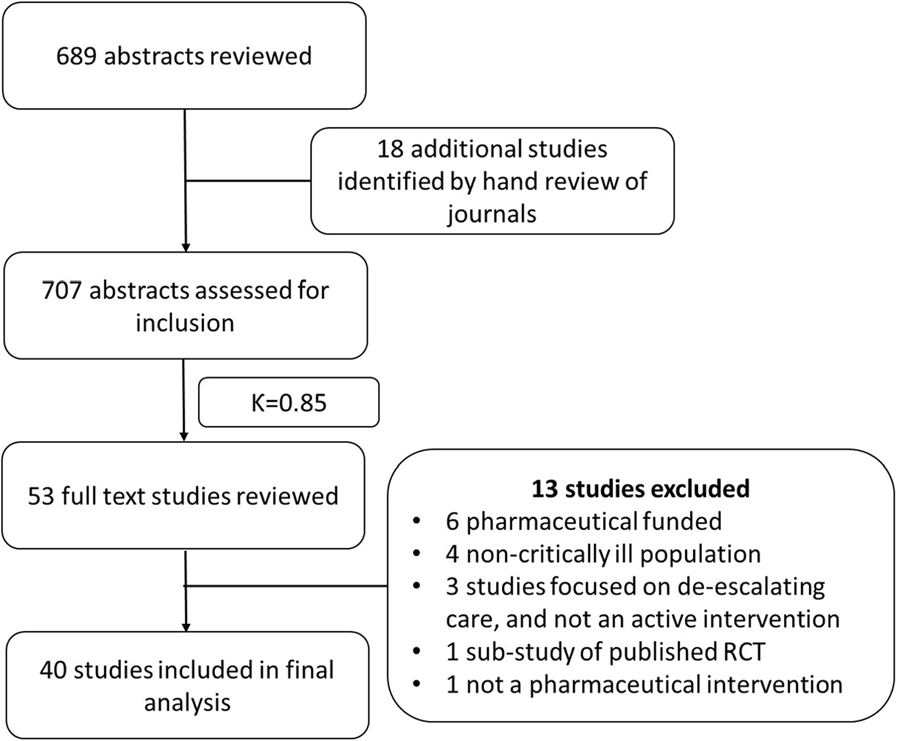
Flow diagram. Kappa reflects agreement among reviewers

A total of 689 abstracts were identified via the search term and an additional 18 abstracts were identified by hand review of the included journals, for a total of 707 abstracts reviewed. Of these, the two abstract reviewers highlighted 53 trials requiring full-text review (*ƙ* = 0.85). After review of these studies in full, 40 studies were ultimately included for full data abstraction. There were no substantial disagreements requiring adjudication by a third reviewer. A total of 40 trials were ultimately included in the analysis. Please see Fig. 1 flow diagram.

### Characteristics of included studies

The included trials represent 28,636 randomized patients with a median of 293 (IQR 100–546) patients per trial. Most studies were blinded (*n* = 34 [85%]) and most were multicenter (*n* = 34 [85%]). A total of 31 (78%) studies had a placebo or usual care control arm. The most common disease states studied were general critical care (35%) and sepsis (25%). JAMA contributed the most studies to the review, 12 (30%). Selected details of the included articles can be found in Supplemental Table 1.

### Adherence to CONSORT harms recommendations and restrictions on AE reporting

Overall, the median number of CONSORT items met was 12 (9–14) of 18. The highest number of items met was 17 and the lowest number was 1. The most commonly met items were “the efficacy and safety analyses were performed using the same populations” (*n* = 36, 90%), “AE results reported separately for each treatment arm” (39, 98%), and “article provides both number of AEs and number of patients with AEs” (37, 93%). Most trials failed to meet items, “article mentions how AE severity grading was performed’ (14, 25%), ‘article lists addressed AEs and provides definitions for each” (19, 48%), “article reports the number of deaths due to AEs in each arm” (18, 45%), “article presents severity grading of AEs” (17, 43%). Please see Table 1 for additional details.

There was no difference in median CONSORT checklist items met comparing multicenter to single-center trials (12 [9–14] vs. 12 [7–14], *p* = 0.79) or blinded to non-blinded trials (12 [9–14] vs. 11 [6–11], *p* = 0.22).

AE reporting was restricted to just serious AEs in 12 (30%) of studies. Eight (20%) studies reported only pre-specified safety outcomes and no studies reported only AEs in which there was a significant difference in AEs between arms.

There were a number of trials examining the same or similar interventions. Three (8%) of the trials examined haloperidol for delirium, four (10%) examined corticosteroids for infection (pneumonia, sepsis, and septic shock), and four (10.0%) examined dexmedetomidine. Among trials of the same or similar intervention, there was substantial variability in the choice, definitions, and rates of safety outcomes and adverse events. Additional details can be found in Tables 2, 3, and 4.

**Table 2:** Reported AEs in haloperidol studies

Study*	Described safety outcomes in the manuscript	Definitions	Events per total patients analyzed	Events per total patients analyzed in intervention arm
1	Extrapyramidal symptoms	According to the modified Simpson Angus Scale	0.01	0.01
	Akathisia	According to 10 cm visual analog scale	Not reported	Not reported
	QTc prolongation	QTc > 500 ms on study day #1 & 2 ECGs or telemetry	0. 15	0.16
	Agitated delirium and oversedation	According to the Richmond Agitation-Sedation Scale	1.28	1.27
	Torsades de Pointes	None provided	0	0
	Drug reaction with eosinophilia	None provided	0	0
23	Extrapyramidal symptoms	Dystonia, tremor, myoclonus, tics, rigidity, akathisia	0.04	0.05
	QTc prolongation	QTc > 500 ms and 10% increase from baseline	0.06	0.06
	Drowsiness	None provided	Not reported	Not reported
36	Extrapyramidal symptoms	Signs of extrapyramidal symptoms measured twice daily	0.02	0.03
	QTc prolongation	QTc ≥ 500 ms or ≥ 60 ms above baseline	0.07	0. 12
	Excessive sedation	As determined by the clinical team	0.02	0.03
	Hypotension	Systolic blood pressure ≤ 90 mmHg)	0.33	0.03

*For the study number reference, please see Supplemental Table 1

**Table 3:** Reported AEs in corticosteroid studies

Study	Described safety outcomes in the manuscript	Definitions	Events per total patients analyzed	Events per total patients analyzed in intervention arm
6	Superinfection	Medical Dictionary for Regulatory Activities classification A new infection ≥ 48 h after randomization up to day 180	0.30	0.31
	Bleeding	Intracranial hemorrhage, any bleeding requiring surgery or at least 2 red blood cell transfusions up to day 28	0.56	0.53
	Gastroduodenal bleeding	None provided	0.07	0.06
	Hyperglycemia	Glucose levels ≥ 150 mg/dL, up to day 7	0.86	0.89
	Neurologic sequelae	Muscular Disability Rating Scale (> 3), up to day 180	0.16	0.18
7	Incidence of new onset bacteremia or fungemia	Between 2 and 14 days, but no definition provided	0.143	0.143
	Blood transfusion	In the ICU, ut no definition provided	0.37	0.39
	Hyperglycemia	None provided	< 0.01	< 0.01
	Hypernatremia	None provided	< 0.01	< 0.01
	Hyperchloremia	None provided	< 0.01	< 0.01
	Hypertension	None provided	< 0.01	< 0.01
	Bleeding	None provided	< 0.01	< 0.01
	Encephalopathy	None provided	< 0.01	< 0.01
	Leukocytosis	None provided	< 0.01	< 0.01
	Myopathy	None provided	< 0.01	< 0.01
	Septic arthritis	None provided	< 0.01	< 0.01
	Ischemic bowel	None provided	< 0.01	< 0.01
	Abdominal-wound dehiscence	None provided	< 0.01	< 0.01
	Circulatory shock	None provided	< 0.01	< 0.01
	Thrombocytopenia	None provided	< 0.01	< 0.01
26	Muscle weakness	Severity of muscle weakness until ICU discharge Evaluated using the Medical research council scale	0.22	0.25
	Weaning failure	Weaning failure is defined as re-intubation within 24 h after extubation OR the need of continuous non-invasive mechanical ventilation with pressure support for more than 48 h after extubation Up to day 28	0.09	0.09
	Secondary infection	Secondary infection is defined as a microbiologically proven new infection which occurs more than 48 h after application of first study medication, with or without adaptation of the antibiotic regime, OR clinical evidence of a new source of infection with or without microbiological verification Up to day 28	0.19	0.22
	Gastrointestinal bleeding	Acute bleeding which requires treatment with more than 1unit of red blood cells within 24 h. Up to day 28	0.01	0.02
	Hypernatremia	Blood sodium level and frequency of hypernatremia (> 155 mmol/l) up to day 14	0.05	0.05
	Hyperglycemia	Blood glucose level and frequency of hyperglycemia (> 150 mg/dl) up to day 14	0.82	0.88
33	Hyperglycemia	None provided	0. 15	0.18
	Superinfection	Patients tested positive for a nosocomial infection of any source.	0.01	0.02
	Gastrointestinal bleeding	None provided	0.01	0
	Delirium	None provided	0.01	0.02
	Acute kidney injury	None provided	0. 13	0. 13
	Acute hepatic failure.	None provided	0.01	0.02

**Table 4:** Reported AEs in dexmedetomidine studies

12	Hypotension	Blood pressure < 90 mmHg	0.27	0.34
	Bradycardia	Heart rate < 50 beats/min	0.09	0. 10
	Hypotension and bradycardia		0.33	0.38
25	Bradycardia	None provided	0.04	0.07
	Acute coronary syndrome	None provided	0.01	0.01
30	Hypotension	Requiring vasopressor support	0	0
	Bradycardia	Requiring interruption of study drug	0	0.03
	Agitation		0.04	0.03
34	Hypoxemia	< 90% or decrease 5% Up to 24 h after surgery	0. 11	0.07
	Bradycardia	(< 55, or decrease > 20% if initial rate < 70 Up to 24 h after surgery	0. 15	0. 17
	Tachycardia	> 160 or if initial rate > 113 then 20% Up to 24 h after surgery	0. 10	0.07
	Hypotension	SBP < 95 of if SBP < 120 then 20%	0.29	0.33
	Hypertension	SBP > 160 or if SBP > 133 then 20% Up to 24 h after surgery	0. 14	0. 10

### Review of protocols

Clinical trial protocols were obtained for 30 (75%) trials. Of these, 25 (80%) protocols provided some definitions for expected AEs. These definitions varied substantially in the level of details provided. Fifteen (50%) protocols provided general definitions for what constitutes a “serious” AE. Six (20%) protocols provided guidance for specific AE severity grading. Three (10%) protocols referenced a validated dictionary (e.g., the Medical Dictionary for Regulated Activities) for AE definition. Sixteen (53%) protocols included guidance for AE attribution to study drugs. In most cases, this guidance was that the site investigator should use the best judgment. In one trial AEs were adjudicated by an independent board.

## Discussion

Published manuscripts for trials included in the present study demonstrated substantial heterogeneity in AE reporting. While some studies adhered closely to CONSORT AE reporting recommendations, many trials included only a few of the recommended elements. Among trials that investigated similar pharmacologic interventions in critically ill populations, AE definitions and incidence varied greatly. Published manuscripts and protocols inconsistently provided guidance for AE severity grading or relatedness attribution.

The 2004 extension to the Consolidated Standards of Reporting Trials (CONSORT) addressed inconsistencies in the reporting of harms-related data from RCTs and introduced a checklist of items to include when reporting trial harms [8]. The aim of this work was to improve harms reporting in RCTs and thereby help front-line clinical providers to better interpret the risks of a studied intervention. While overall reporting of RCT harms seems to have improved following the publication of the CONSORT extension, harms reporting in many trials across medical disciplines remains insufficient [9].

A number of studies have explored adherence to CONSORT AE reporting recommendations in non-critically ill patient cohorts. These populations have included cardiovascular health [10], urology [11], epilepsy [6], alternative medical therapies [12], and various mixed populations [13, 14] In one systematic review, there was substantial heterogeneity in adherence to various CONSORT harms reporting checklist items [15]. In the present study of trials conducted in the critically ill population, we likewise found that adherence to many important CONSORT AE reporting items was low. Less than half of the trials we reviewed provided clear AE definitions, explained how AEs were attributed to study drug, or presented data on AE severity. Further, even when AE reporting was done according to CONSORT standards, the information was commonly only obtainable through a detailed review of the trial’s supplementary data or the study protocol.

Attribution of AEs to study drugs in critically ill populations is challenging given the sometimes rapidly progressive and severe nature of the diseases studied. The US Food and Drug Administration defines a suspected adverse reaction as one in which there is a “reasonable possibility of drug related causality,” but does not specify what degree of confidence is required or how that degree of confidence should be arrived at (Code of Federal Regulations: 21CFR312.32). In Europe, a suspected AE reflects “a reasonable possibility (defined as the presence of facts or arguments to support) of a causal relationship between the event and the investigational product.” [16] In the present study, we found that published protocols uncommonly provided information on how attribution was assessed. Even in the review of the study protocols, which often contain substantially more detailed information on trial procedures, there was frequently no guidance for AE attribution. In cases where guidance was provided, investigators were generally asked to rely on their intuition.

Several of the most commonly reported AEs in the included trials (e.g., electrolyte abnormalities, bradycardia, hypotension, and superinfections) are inherent to the disease state and the non-research interventions applied in the ICU. Hence, distinctions between the progression of the disease and AEs are difficult if not impossible even if guidance for AE attribution is provided. An illustrative example is the APROCCHSS and ADRENAL trials, both investigating the effects of glucocorticoid therapy in patients with septic shock [17, 18]. In the APROCCHSS trial, 1645 AEs were recorded in 1241 patients while the ADRENAL trial reported 33 AEs in 3658 patients. Although the mortality was higher in the APROCCHSS trial and the APROCCHSS trial included some patients who received drotrecogin alpha, the large difference in rates of safety outcomes between the two studies is likely to be related to differences in reporting and not actual differences in rates of AEs related to corticosteroids. In the ADRENAL trial, it was stated that “reporting of adverse events will be restricted to events that are considered to be related to study treatment (possibly, probably or definitely).” [17] As above, determining relatedness of AEs in a critically ill patient population is challenging—especially when investigators are asked to rely on intuition alone.

Given the inherent subjectivity of reliance on investigator intuition when evaluating AE relatedness [19], other specialties are moving towards more structured attribution processes including the use of standardized scales such as the Naranjo Scale, which aims to standardize the assessment of causality for AEs through a series of questions answered by the AE adjudicator [20, 21].

Severity grading of AEs was often not performed in the reviewed trials, and when it was performed, severity grading rarely followed any common scale. This was especially striking when AE reporting was reviewed for different studies of the same intervention. While official authorities do provide some guidance on AE severity grading, this guidance is generally aimed at trials conducted primarily in the outpatient setting and is primarily oriented towards clinical trials of cancer therapies [22, 23]. Critically ill patients often suffer from more severe organ dysfunction at the time of enrollment and traditional outpatient-directed severity grading scales may not apply.

A commentary by Cook et al. identified five major challenges that are specific to AE reporting for clinical trials in critical care populations [3]. These challenges include how to define serious AEs, how to interpret AEs in light of the natural progression of critical illness, how to attribute AEs to drugs being tested, how to determine whether death is related to study drug, and how trial monitoring boards can interpret serious adverse events as the trial enrolls. Solutions to these problems may also require creative critical-care focused approaches, such as identifying and defining (including attribution processes and severity grading) the most concerning expected adverse events a priori to allow for a more focused review of cases unfolding upon the backdrop of a critical illness. Taking this a step further, the difficulties with attribution in critical care trials may in fact require investigators to simply report the number of a priori defined adverse events in each group and assess for significant differences between arms. This, however, is complicated where trials are powered to a primary outcome, but are not powered to detect differences in sometimes rare events. Meta-analyses may allow pooling of AE rates and therefore increased power to detect differences; however, this would require standardization of definitions and monitoring procedures for AEs across trials.

To our knowledge, this is the first study to systematically study harms collection and reporting in trials conducted within critically ill populations. In addition, the inclusion of study protocol review allowed for a more thorough understanding of harms collection in these trials. This trial had a number of limitations. First, this study includes a relatively small number of trials, although the included trials do represent a contemporary sampling. Second, only articles published in eight high-impact journals were included and the results presented might not be reflective of all critical care publications. The choice of journals to review carried some subjectivity. Third, given that review of CONSORT items included some degree of subjectivity, we strove to be as lenient as possible in our assessment of harms reporting which may have overestimated trial performance on the CONSORT checklist. Finally, all CONSORT Harms items in this trial were given equal weighting in the analysis, but it could be argued that some items should carry more weight than others.

## Conclusions

Randomized trials of pharmacologic interventions conducted in critically ill populations and published in high impact journals often fail to adequately describe AE definitions, severity, attribution, and collection procedures. Among trials of similar interventions in comparable populations, variation in AE collection and reporting procedures is substantial. These factors may limit a clinician’s ability to accurately balance the potential benefits and harms of an intervention.

## Supplementary information

**Additional file 1.**

## Data Availability

The datasets used and/or analyzed during the current study are available from the corresponding author on reasonable request.

## Abbreviations

CONSORT: Consolidated Standards of Reporting Trials
AE: Adverse event
ICU: Intensive care unit
RCT: Randomized control trial
PRISMA: Preferred Reporting Items for Systematic Reviews and Meta-Analyses
