# Supplementary material for: Identification, collection, and reporting of harms among non-industry-sponsored randomized clinical trials of pharmacologic interventions in the critically ill population: a systematic review
Source: Crit Care. 2020 Jul 8;24:398. doi: 10.1186/s13054-020-03113-z (PMC7346341; doi:10.1186/s13054-020-03113-z)
Supplement: Supplementary file 1 — Additional file 1. [file 13054_2020_3113_MOESM1_ESM.docx]

**SUPPLEMENTARY MATERIALS**

**Contents**

[Search Terms 1](#_Toc41687487)

[Supplementary Table #1: Articles included in Analysis 2](#_Toc41687488)

[References 4](#_Toc41687489)

**Search Terms**

("critical illness"[MeSH Terms] OR critical illness[TIAB] OR critically ill[TIAB] OR "critical care"[MeSH Terms] OR critical care[TIAB] OR intensive care[TIAB] OR "intensive care units"[MeSH Terms] OR intensive care unit[TIAB] OR "sepsis"[MeSH Terms] OR sepsis[TIAB] AND "shock, septic"[MeSH Terms] OR septic shock[TIAB] OR "acute lung injury"[MeSH Terms] OR acute lung injury[TIAB] OR "respiratory distress syndrome, Adult"[MeSH Terms] OR "respiratory insufficiency"[MeSH Terms] OR ARDS[TIAB] OR "Resuscitation"[MeSH Terms] OR resuscitation[TIAB] OR "Shock"[MeSH Terms] OR "heart arrest"[MeSH Terms] OR cardiac arrest[TIAB] OR "death, sudden, cardiac"[MeSH Terms] OR "cardiopulmonary resuscitation"[MeSH Terms] OR cardiopulmonary arrest[TIAB] OR "delirium"[MeSH Terms] OR "gastrointestinal hemorrhage"[MeSH Terms] OR "brain injuries, traumatic"[MeSH Terms] OR "stroke"[MeSH Terms] OR "cerebral hemorrhage"[MeSH Terms] OR "pulmonary embolism"[MeSH Terms] OR "pancreatitis"[MeSH Terms] OR "shock, cardiogenic"[MeSH Terms] OR "shock, hemorrhagic"[MeSH Terms])

AND

(randomized controlled trial[PT] OR controlled clinical trial[PT] OR randomized[TIAB] OR placebo[TIAB] OR "drug therapy"[Subheading] OR randomly[TIAB] OR trial[TIAB] OR groups[TIAB] NOT ("animals"[MeSH Terms] NOT "humans"[MeSH Terms]))

AND

("The New England Journal of Medicine"[Journal] OR "Lancet (London, England)"[Journal] OR "Journal of the American Medical Association"[Journal] OR “JAMA” [Journal] OR "British Medical Journal"[Journal] OR “BMJ” [Journal] OR "Critical Care Medicine"[Journal] OR "Intensive Care Medicine"[Journal] OR "Chest"[Journal] OR "American Journal of Respiratory and Critical Care Medicine"[Journal])

AND

English[LA] AND ("2015/01/01"[PDAT] : "2018/12/31"[PDAT])

NOT

(observational study[PT] OR case reports[PT] OR letter[PT] OR comment[PT] OR editorial[PT])

# **Supplementary Table #1: Articles included in Analysis**

| **Manuscript #** | **Year** | **Journal** | **Region** | **Multi-center** | **Patients** | **Disease state** | **Blinded** | **Intervention(s)** | **Comparator(s)** |
| --- | --- | --- | --- | --- | --- | --- | --- | --- | --- |
| [1] | Haloperidol and Ziprasidone for Treatment of Delirium in Critical Illness | | | | | | | | |
|  | 2018 | NEJM | North America | Yes | 566 | Delirium | Yes | 1) Haloperidol  2) Ziprasodone | Placebo |
| [2] | Pantoprazole in Patients at Risk for Gastrointestinal Bleeding in the ICU | | | | | | | | |
|  | 2018 | NEJM | Europe | Yes | 3282 | General critical care | yes | Pantoprazole | Placebo |
| [3] | Restricted fluid resuscitation in suspected sepsis associated hypotension (REFRESH): a pilot randomised controlled trial | | | | | | | | |
|  | 2018 | ICM | Australia/NZ | Yes | 99 | Sepsis | No | Early vasopressor | Intravenous fluids |
| [4] | Terlipressin versus norepinephrine as infusion in patients with septic shock: a multicentre, randomised, double-blinded trial | | | | | | | | |
|  | 2018 | ICM | Asia | Yes | 526 | Sepsis | yes | Terlipressin | Norepinephrine |
| [5] | Efficacy and Safety of Combination Therapy of Shenfu Injection and Postresuscitation Bundle in Patients With Return of Spontaneous Circulation After In-Hospital Cardiac Arrest: A Randomized, Assessor-Blinded, Controlled Trial | | | | | | | | |
|  | 2017 | CCM | Asia | Yes | 978 | Cardiac Arrest | yes | Shenfu | Placebo |
| [6] | Hydrocortisone plus Fludrocortisone for Adults with Septic Shock | | | | | | | | |
|  | 2018 | NEJM | Europe | Yes | 1241 | Sepsis | yes | Hydrocortisone  & fludrocortisone | Placebo |
| [7] | Adjunctive Glucocorticoid Therapy in Patients with Septic Shock | | | | | | | | |
|  | 2018 | NEJM | Australia/NZ | Yes | 3658 | Sepsis | yes | Hydrocortisone infusion | Placebo |
| [8] | Levosimendan for Hemodynamic Support after Cardiac Surgery | | | | | | | | |
|  | 2017 | NEJM | Europe | Yes | 506 | Shock | yes | Levosimendan | Placebo |
| [9] | Randomized Clinical Trial of a Combination of an Inhaled Corticosteroid and Beta Agonist in Patients at Risk of Developing the Acute Respiratory Distress Syndrome | | | | | | | | |
|  | 2017 | CCM | North America | Yes | 60 | ARDS | yes | Aerosolized inhaled corticosteroid and beta-agonist | Placebo |
| [10] | Sevoflurane for Sedation in Acute Respiratory Distress Syndrome. A Randomized Controlled Pilot Study | | | | | | | | |
|  | 2017 | AJRCCM | Europe | No | 50 | ARDS | no | Sevoflurane | Midazolam |
| [11] | Thrombolytic removal of intraventricular haemorrhage in treatment of severe stroke: results of the randomised, multicentre, multiregion, placebo-controlled CLEAR III trial | | | | | | | | |
|  | 2017 | Lancet | North America | Yes | 500 | General critical care | yes | Alteplase | Saline |
| [12] | Low-Dose Nocturnal Dexmedetomidine Prevents ICU Delirium. A Randomized, Placebo-controlled Trial | | | | | | | | |
|  | 2018 | AJRCCM | North America | Yes | 100 | Delirium | yes | Dexmedetomidine | Placebo |
| [13] | A Multicenter Randomized Trial of Continuous versus Intermittent β-Lactam Infusion in Severe Sepsis | | | | | | | | |
|  | 2015 | AJRCCM | Australia/NZ | Yes | 432 | Sepsis | yes | Continuous B-lactam infusion | Intermittent B-lactam infusion |
| [14] | Effect of Administration of Ramelteon, a Melatonin Receptor Agonist, on the Duration of Stay in the ICU: A Single-Center Randomized Placebo-Controlled Trial | | | | | | | | |
|  | 2018 | CCM | Asia | No | 88 | Delirium | yes | Ramelteon | Placebo |
| [15] | Nebulized Versus IV Amikacin as Adjunctive Antibiotic for Hospital and Ventilator-Acquired Pneumonia Postcardiac Surgeries: A Randomized Controlled Trial | | | | | | | | |
|  | 2018 | CCM | Africa | No | 133 | General critical care | no | Nebulized amikacin | Intravenous amikacin |
| [16] | Intravenous iron or placebo for anaemia in intensive care: the IRONMAN multicentre randomized blinded trial : A randomized trial of IV iron in critical illness | | | | | | | | |
|  | 2016 | ICM | Australia/NZ | Yes | 140 | General critical care | yes | Intravenous iron | Placebo |
| [17] | Erythromycin versus metoclopramide for post-pyloric spiral nasoenteric tube placement: a randomized non-inferiority trial | | | | | | | | |
|  | 2018 | ICM | Asia | Yes | 332 | General critical care | no | Erythromycin | Metoclopramide |
| [18] | Acetaminophen for Fever in Critically Ill Patients with Suspected Infection | | | | | | | | |
|  | 2015 | NEJM | Australia/NZ | Yes | 690 | General critical care | yes | Acetaminophen | Placebo |
| [19] | Levosimendan for the Prevention of Acute Organ Dysfunction in Sepsis | | | | | | | | |
|  | 2016 | NEJM | Europe | Yes | 515 | Shock | yes | Levosimendan | Placebo |
| [20] | Erythropoietin in Traumatic Brain Injury (EPO-TBI): a double-blind randomised controlled trial | | | | | | | | |
|  | 2015 | Lancet | Australia/NZ | Yes | 596 | Trauma | yes | Erythropoietin | Placebo |
| [21] | A Randomized Trial of the Amikacin Fosfomycin Inhalation System for the Adjunctive Therapy of Gram-Negative Ventilator-Associated  Pneumonia: IASIS Trial | | | | | | | | |
|  | 2017 | Chest | North America | Yes | 142 | General critical care | yes | Amikacin/fosfomycin inhalation | Placebo |
| [22] | Decontamination Strategies and Bloodstream Infections With Antibiotic-Resistant Microorganisms in Ventilated Patients: A Randomized Clinical Trial | | | | | | | | |
|  | 2018 | JAMA | Europe | Yes | 8496 | General critical care | no | CHX/SDE/SOD | Baseline usual care |
| [23] | Effect of Haloperidol on Survival Among Critically Ill Adults With a High Risk of Delirium: The REDUCE Randomized Clinical Trial | | | | | | | | |
|  | 2018 | JAMA | Europe | Yes | 1789 | Delirium | yes | Haloperidol | Placebo |
| [24] | Effect of Ganciclovir on IL-6 Levels Among Cytomegalovirus-Seropositive Adults With Critical Illness: A Randomized Clinical Trial | | | | | | | | |
|  | 2017 | JAMA | North America | Yes | 156 | General critical care | yes | Gancyclovir | Placebo |
| [25] | Effect of Dexmedetomidine on Mortality and Ventilator-Free Days in Patients Requiring Mechanical Ventilation With Sepsis: A Randomized Clinical Trial | | | | | | | | |
|  | 2017 | JAMA | Asia | Yes | 201 | Sepsis | yes | Dexmedetomidine | Placebo |
| [26] | Effect of Hydrocortisone on Development of Shock Among Patients With Severe Sepsis: The HYPRESS Randomized Clinical Trial | | | | | | | | |
|  | 2016 | JAMA | Europe | Yes | 353 | Sepsis | yes | Hydrocortisone | Placebo |
| [27] | Empirical Micafungin Treatment and Survival Without Invasive Fungal Infection in Adults With ICU-Acquired Sepsis, Candida Colonization, and Multiple Organ Failure: The EMPIRICUS Randomized Clinical Trial | | | | | | | | |
|  | 2016 | JAMA | Europe | Yes | 251 | Sepsis | yes | Micafungin | Placebo |
| [28] | Effect of Early Vasopressin vs Norepinephrine on Kidney Failure in Patients With Septic Shock: The VANISH Randomized Clinical Trial | | | | | | | | |
|  | 2016 | JAMA | Europe | Yes | 408 | Sepsis | yes | Vasopressin, hydrocortisone | Norepinephrine |
| [29] | Effect of Aspirin on Development of ARDS in At-Risk Patients Presenting to the Emergency Department: The LIPS-A Randomized Clinical Trial | | | | | | | | |
|  | 2016 | JAMA | North America | Yes | 390 | ARDS | yes | ASA | Placebo |
| [30] | Effect of Dexmedetomidine Added to Standard Care on Ventilator-Free Time in Patients With Agitated Delirium: A Randomized Clinical Trial | | | | | | | | |
|  | 2016 | JAMA | Australia/NZ | Yes | 71 | Delirium | yes | Dexmedetomidine | Placebo |
| [31] | Effect of Inhaled Xenon on Cerebral White Matter Damage in Comatose Survivors of Out-of-Hospital Cardiac Arrest: A Randomized Clinical Trial | | | | | | | | |
|  | 2016 | JAMA | Europe | Yes | 97 | Cardiac Arrest | yes | Xenon | Placebo |
| [32] | Effect of Acetazolamide vs Placebo on Duration of Invasive Mechanical Ventilation Among Patients With Chronic Obstructive Pulmonary Disease: A Randomized Clinical Trial | | | | | | | | |
|  | 2016 | JAMA | Europe | Yes | 380 | General critical care | yes | Acetazolamide | Placebo |
| [33] | Effect of corticosteroids on treatment failure among hospitalized patients with severe community-acquired pneumonia and high inflammatory response: a randomized clinical trial | | | | | | | | |
|  | 2015 | JAMA | Europe | Yes | 120 | Sepsis | yes | Corticosteroids | Placebo |
| [34] | Dexmedetomidine for prevention of delirium in elderly patients after non-cardiac surgery: a randomised, double-blind, placebo-controlled trial | | | | | | | | |
|  | 2016 | Lancet | Asia | Yes | 700 | Delirium | yes | Precedex | Placebo |
| [35] | Pantoprazole or Placebo for Stress Ulcer Prophylaxis (POP-UP): Randomized Double-Blind Exploratory Study | | | | | | | | |
|  | 2016 | Critical care medicine | Australia/NZ | No | 209 | General critical care | yes | Pantoprazole | Placebo |
| [36] | Preventing ICU Subsyndromal Delirium Conversion to Delirium With Low-Dose IV Haloperidol: A Double-Blind, Placebo-Controlled Pilot Study | | | | | | | | |
|  | 2016 | Critical care medicine | North America | No | 68 | Delirium | yes | Haloperidol | Placebo |
| [37] | Randomized, Double-Blind, Placebo-Controlled Trial of Thiamine as a Metabolic Resuscitator in Septic Shock: A Pilot Study | | | | | | | | |
|  | 2016 | Critical care medicine | North America | Yes | 88 | Sepsis | yes | Thiamine | Placebo |
| [38] | Fentanyl as pre-emptive treatment of pain associated with turning mechanically ventilated patients: a randomized controlled feasibility study | | | | | | | | |
|  | 2016 | Intensive care medicine | Europe | Yes | 75 | General critical care | yes | Fentanyl | Placebo |
| [39] | Inhaled nitric oxide for acute chest syndrome in adult sickle cell patients: a randomized controlled study | | | | | | | | |
|  | 2015 | Intensive care medicine | Europe | Yes | 100 | ARDS | yes | iNO | Placebo |
| [40] | A Randomized Study of a Single Dose of Intramuscular Cholecalciferol in Critically Ill Adults | | | | | | | | |
|  | 2015 | Critical care medicine | Australia/NZ | No | 50 | General critical care | no | High dose cholecalciferol | Low dose cholecalciferol |

**References**

1. Girard, T.D., et al., *Haloperidol and Ziprasidone for Treatment of Delirium in Critical Illness.* N Engl J Med, 2018. **379**(26): p. 2506-2516.

2. Krag, M., et al., *Pantoprazole in Patients at Risk for Gastrointestinal Bleeding in the ICU.* N Engl J Med, 2018. **379**(23): p. 2199-2208.

3. Macdonald, S.P.J., et al., *Restricted fluid resuscitation in suspected sepsis associated hypotension (REFRESH): a pilot randomised controlled trial.* Intensive Care Med, 2018. **44**(12): p. 2070-2078.

4. Liu, Z.M., et al., *Terlipressin versus norepinephrine as infusion in patients with septic shock: a multicentre, randomised, double-blinded trial.* Intensive Care Med, 2018. **44**(11): p. 1816-1825.

5. Zhang, Q., et al., *Efficacy and Safety of Combination Therapy of Shenfu Injection and Postresuscitation Bundle in Patients With Return of Spontaneous Circulation After In-Hospital Cardiac Arrest: A Randomized, Assessor-Blinded, Controlled Trial.* Crit Care Med, 2017. **45**(10): p. 1587-1595.

6. Annane, D., et al., *Hydrocortisone plus Fludrocortisone for Adults with Septic Shock.* N Engl J Med, 2018. **378**(9): p. 809-818.

7. Venkatesh, B., et al., *Adjunctive Glucocorticoid Therapy in Patients with Septic Shock.* N Engl J Med, 2018. **378**(9): p. 797-808.

8. Landoni, G., et al., *Levosimendan for Hemodynamic Support after Cardiac Surgery.* N Engl J Med, 2017. **376**(21): p. 2021-2031.

9. Festic, E., et al., *Randomized Clinical Trial of a Combination of an Inhaled Corticosteroid and Beta Agonist in Patients at Risk of Developing the Acute Respiratory Distress Syndrome.* Crit Care Med, 2017. **45**(5): p. 798-805.

10. Jabaudon, M., et al., *Sevoflurane for Sedation in Acute Respiratory Distress Syndrome. A Randomized Controlled Pilot Study.* Am J Respir Crit Care Med, 2017. **195**(6): p. 792-800.

11. Hanley, D.F., et al., *Thrombolytic removal of intraventricular haemorrhage in treatment of severe stroke: results of the randomised, multicentre, multiregion, placebo-controlled CLEAR III trial.* Lancet, 2017. **389**(10069): p. 603-611.

12. Skrobik, Y., et al., *Low-Dose Nocturnal Dexmedetomidine Prevents ICU Delirium. A Randomized, Placebo-controlled Trial.* Am J Respir Crit Care Med, 2018. **197**(9): p. 1147-1156.

13. Dulhunty, J.M., et al., *A Multicenter Randomized Trial of Continuous versus Intermittent β-Lactam Infusion in Severe Sepsis.* Am J Respir Crit Care Med, 2015. **192**(11): p. 1298-305.

14. Nishikimi, M., et al., *Effect of Administration of Ramelteon, a Melatonin Receptor Agonist, on the Duration of Stay in the ICU: A Single-Center Randomized Placebo-Controlled Trial.* Crit Care Med, 2018. **46**(7): p. 1099-1105.

15. Hassan, N.A., et al., *Nebulized Versus IV Amikacin as Adjunctive Antibiotic for Hospital and Ventilator-Acquired Pneumonia Postcardiac Surgeries: A Randomized Controlled Trial.* Crit Care Med, 2018. **46**(1): p. 45-52.

16. Litton, E., et al., *Intravenous iron or placebo for anaemia in intensive care: the IRONMAN multicentre randomized blinded trial : A randomized trial of IV iron in critical illness.* Intensive Care Med, 2016. **42**(11): p. 1715-1722.

17. Hu, B., et al., *Erythromycin versus metoclopramide for post-pyloric spiral nasoenteric tube placement: a randomized non-inferiority trial.* Intensive Care Med, 2018. **44**(12): p. 2174-2182.

18. Young, P., et al., *Acetaminophen for Fever in Critically Ill Patients with Suspected Infection.* N Engl J Med, 2015. **373**(23): p. 2215-24.

19. Gordon, A.C., et al., *Levosimendan for the Prevention of Acute Organ Dysfunction in Sepsis.* N Engl J Med, 2016. **375**(17): p. 1638-1648.

20. Nichol, A., et al., *Erythropoietin in traumatic brain injury (EPO-TBI): a double-blind randomised controlled trial.* Lancet, 2015. **386**(10012): p. 2499-506.

21. Kollef, M.H., et al., *A Randomized Trial of the Amikacin Fosfomycin Inhalation System for the Adjunctive Therapy of Gram-Negative Ventilator-Associated Pneumonia: IASIS Trial.* Chest, 2017. **151**(6): p. 1239-1246.

22. Wittekamp, B.H., et al., *Decontamination Strategies and Bloodstream Infections With Antibiotic-Resistant Microorganisms in Ventilated Patients: A Randomized Clinical Trial.* JAMA, 2018. **320**(20): p. 2087-2098.

23. van den Boogaard, M., et al., *Effect of Haloperidol on Survival Among Critically Ill Adults With a High Risk of Delirium: The REDUCE Randomized Clinical Trial.* JAMA, 2018. **319**(7): p. 680-690.

24. Limaye, A.P., et al., *Effect of Ganciclovir on IL-6 Levels Among Cytomegalovirus-Seropositive Adults With Critical Illness: A Randomized Clinical Trial.* JAMA, 2017. **318**(8): p. 731-740.

25. Kawazoe, Y., et al., *Effect of Dexmedetomidine on Mortality and Ventilator-Free Days in Patients Requiring Mechanical Ventilation With Sepsis: A Randomized Clinical Trial.* JAMA, 2017. **317**(13): p. 1321-1328.

26. Keh, D., et al., *Effect of Hydrocortisone on Development of Shock Among Patients With Severe Sepsis: The HYPRESS Randomized Clinical Trial.* JAMA, 2016. **316**(17): p. 1775-1785.

27. Timsit, J.F., et al., *Empirical Micafungin Treatment and Survival Without Invasive Fungal Infection in Adults With ICU-Acquired Sepsis, Candida Colonization, and Multiple Organ Failure: The EMPIRICUS Randomized Clinical Trial.* JAMA, 2016. **316**(15): p. 1555-1564.

28. Gordon, A.C., et al., *Effect of Early Vasopressin vs Norepinephrine on Kidney Failure in Patients With Septic Shock: The VANISH Randomized Clinical Trial.* JAMA, 2016. **316**(5): p. 509-18.

29. Kor, D.J., et al., *Effect of Aspirin on Development of ARDS in At-Risk Patients Presenting to the Emergency Department: The LIPS-A Randomized Clinical Trial.* JAMA, 2016. **315**(22): p. 2406-14.

30. Reade, M.C., et al., *Effect of Dexmedetomidine Added to Standard Care on Ventilator-Free Time in Patients With Agitated Delirium: A Randomized Clinical Trial.* JAMA, 2016. **315**(14): p. 1460-8.

31. Laitio, R., et al., *Effect of Inhaled Xenon on Cerebral White Matter Damage in Comatose Survivors of Out-of-Hospital Cardiac Arrest: A Randomized Clinical Trial.* JAMA, 2016. **315**(11): p. 1120-8.

32. Faisy, C., et al., *Effect of Acetazolamide vs Placebo on Duration of Invasive Mechanical Ventilation Among Patients With Chronic Obstructive Pulmonary Disease: A Randomized Clinical Trial.* JAMA, 2016. **315**(5): p. 480-8.

33. Torres, A., et al., *Effect of corticosteroids on treatment failure among hospitalized patients with severe community-acquired pneumonia and high inflammatory response: a randomized clinical trial.* JAMA, 2015. **313**(7): p. 677-86.

34. Su, X., et al., *Dexmedetomidine for prevention of delirium in elderly patients after non-cardiac surgery: a randomised, double-blind, placebo-controlled trial.* Lancet, 2016. **388**(10054): p. 1893-1902.

35. Selvanderan, S.P., et al., *Pantoprazole or Placebo for Stress Ulcer Prophylaxis (POP-UP): Randomized Double-Blind Exploratory Study.* Crit Care Med, 2016. **44**(10): p. 1842-50.

36. Al-Qadheeb, N.S., et al., *Preventing ICU Subsyndromal Delirium Conversion to Delirium With Low-Dose IV Haloperidol: A Double-Blind, Placebo-Controlled Pilot Study.* Crit Care Med, 2016. **44**(3): p. 583-91.

37. Donnino, M.W., et al., *Randomized, Double-Blind, Placebo-Controlled Trial of Thiamine as a Metabolic Resuscitator in Septic Shock: A Pilot Study.* Crit Care Med, 2016. **44**(2): p. 360-7.

38. Robleda, G., et al., *Fentanyl as pre-emptive treatment of pain associated with turning mechanically ventilated patients: a randomized controlled feasibility study.* Intensive Care Med, 2016. **42**(2): p. 183-91.

39. Maitre, B., et al., *Inhaled nitric oxide for acute chest syndrome in adult sickle cell patients: a randomized controlled study.* Intensive Care Med, 2015. **41**(12): p. 2121-9.

40. Nair, P., et al., *A Randomized Study of a Single Dose of Intramuscular Cholecalciferol in Critically Ill Adults.* Crit Care Med, 2015. **43**(11): p. 2313-20.
